# Estimation of Lower Limb Joint Angles Using sEMG Signals and RGB-D Camera

**DOI:** 10.3390/bioengineering11101026

**Published:** 2024-10-15

**Authors:** Guoming Du, Zhen Ding, Hao Guo, Meichao Song, Feng Jiang

**Affiliations:** 1 School of Computer Science and Technology, Harbin Institute of Technology, Harbin 150001, China; 19b903025@stu.hit.edu.cn (G.D.); guohao@hit.edu.cn (H.G.); 23b936020@stu.hit.edu.cn (M.S.); 2 College of Computer and Control Engineering, Northeast Forestry University, Harbin 150040, China; dingzhen@nefu.edu.cn

**Keywords:** joint angle estimation, sEMG, RGB-D camera, dual-branch convolutional network

## Abstract

Estimating human joint angles is a crucial task in motion analysis, gesture recognition, and motion intention prediction. This paper presents a novel model-based approach for generating reliable and accurate human joint angle estimation using a dual-branch network. The proposed network leverages combined features derived from encoded sEMG signals and RGB-D image data. To ensure the accuracy and reliability of the estimation algorithm, the proposed network employs a convolutional autoencoder to generate a high-level compression of sEMG features aimed at motion prediction. Considering the variability in the distribution of sEMG signals, the proposed network introduces a vision-based joint regression network to maintain the stability of combined features. Taking into account latency, occlusion, and shading issues with vision data acquisition, the feature fusion network utilizes high-frequency sEMG features as weights for specific features extracted from image data. The proposed method achieves effective human body joint angle estimation for motion analysis and motion intention prediction by mitigating the effects of non-stationary sEMG signals.

## 1. Introduction

Human–machine interaction (HMI) technology has consistently been a promising domain in automation, healthcare, and other fields of human activities for decades. To conduct a quantitative analysis of human motions and activities during HMI, human body joint angles and positions are utilized as essential metrics. Human activities can be captured and analyzed using wearable devices [1,2,3,4] and vision-based sensors [5].

In-depth analysis of human activity allows for joint angles to be extracted during movements, providing key information for motion pattern detection, movement recognition, and prediction. For example, Da Silva et al. [6] utilized an FBG sensing glove to achieve continuous hand posture reconstruction through joint angles. Kim J. S. et al. [7] enhanced the accuracy and applicability of fiber Bragg grating (FBG) sensors and proposed a real-time tracking algorithm for hand joints. Although strain-based sensors can provide low-latency data for posture, similar applications for larger body parts such as the knee joint and elbow joint are less convenient. Joint axis and position identification has proven effective for joint angle measurement using inertial measurement unit (IMU) sensors [8,9,10], leading to improved accuracy and flexibility in higher-level applications such as gait analysis [8] and activity recognition [11]. Signals captured by surface electromyographic (sEMG) equipment are more sensitive to variations in muscle actions. Consequently, motion models for primary human limbs, such as arms [12] and legs [13,14], yield less invariant results across individuals. Furthermore, the time-advanced features of sEMG signals in human motion provide opportunities for human motion prediction [15,16,17,18].

Compared to wearable devices, sensors such as optical cameras, laser-based cameras, and electromagnetic field sensing devices offer greater flexibility. Gu et al. [19] used a single RGB camera to conduct gait analysis via 2D joint angular information. More detailed 3D joint features can be achieved using red green blue and depth (RGB-D) cameras, yielding outstanding results [20,21]. Radar-based sensing and analysis have achieved promising outcomes in human posture detection [22,23,24], but the relatively higher errors in joint location estimation limit their precise application. As convolutional neural networks become increasingly integrated with image processing technologies, predicting and generating redundant data for joint estimation have become more feasible. Mehta et al. [25] presented a novel approach for 3D motion sensing using a single RGB camera. This approach extends to multi-individual analysis due to the wide sensing field of wear-less sensors. Wear-less sensors have achieved excellent results in joint angle estimation and motion analysis, but disadvantages such as occlusions and insufficient resolutions remain challenges that need to be mitigated.

Attempts to use fused data for angle estimation have been made to improve the accuracy of results and alleviate misrecognition caused by sensor limitations. Filter-based algorithms function effectively with the classical combination of IMU and vision systems [26], while Bo et al. [27] aided in rehabilitation using a combination of depth cameras and inertial sensors. With network techniques, different types of sensing fusion methods offer more options for pose estimation [28,29,30]. To address real-time online processing, synchronization of multiple sensors is a critical issue. The hysteresis in capture timing and varying signal window durations may cause cumulative regression. Data fusion plays a key role in training and processing. Cippitelli et al. [31] achieved relatively precise synchronization of depth cameras and IMUs through auxiliary communication nodes. Processing embedded data remains a challenging issue in computer science, as multiple data sources are handled in both raw and processed forms [32,33]. In human motion recognition applications, rapid information fusion is required without losing detailed semantic-level features.

Achieving accurate human joint angle estimation necessitates addressing individual variations as a key challenge. These variations are caused by differences in physical body structure, movement behaviors, sensor placements, environmental conditions, and other factors. Optimal sensor utilization is an effective approach to improving robustness and adaptability in these complex conditions. Vibrations in sEMG signals introduce noise patterns, which can have significant effects during the processing stage. Wavelet transform techniques on a timescale can mitigate this effect [33], but the loss of detailed information may also decrease the accuracy of joint angle regression and motion pattern recognition. Introducing vision-based sensors and processing techniques enhances data diversity, thereby strengthening stability across different scenarios. The speed of computation is critical in human motion-related applications; tools like encoder–decoder [34,35] and fast convolutional [36,37] operations can provide timely and effective feature extraction.

This paper aims to improve the accuracy and stability of human joint angle estimation for applications in human motion analysis. The contributions of this work can be summarized as follows:

1. Our work presents a novel dual-branch framework for human joint angle estimation that leverages sEMG signals and RGB-D camera data. The multi-dimensional feature extraction block offers a more comprehensive representation of the human joint model across different scenarios.
2. This paper proposes an enhanced sEMG feature extraction method that utilizes multiple channels and scales of sEMG signals through an online adaptive convolutional autoencoder structure, coupled with feature enhancement based on correlation principles.
3. We employ a feature extraction and fusion pipeline that combines sEMG signals and RGB-D data, which has proven effective and efficient in convolutional networks by leveraging multi-scale input and a sensitive data fusion block.

The structure of this paper is as follows. The design of human joint angle estimation is presented in Section 2. The experimental verification of the framework in this paper is described in Section 3. The discussion appears in Section 4 and conclusions are presented in Section 5.

## 2. Materials and Methods

### 2.1. Overview

**Figure 1:**
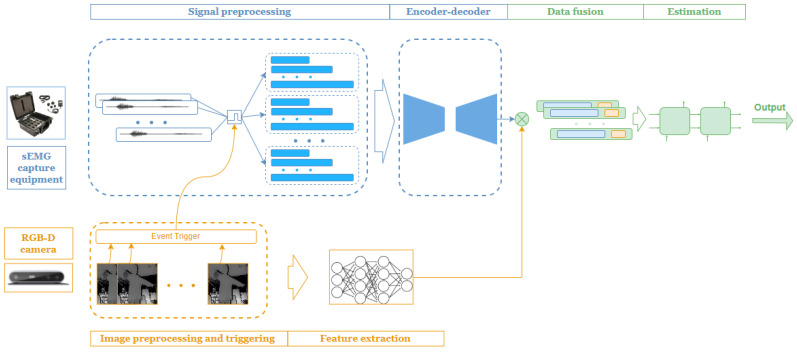
Global framework.

The proposed dual-branch framework is briefly described in Figure 1. The processing system mainly consists of three components: the sEMG signal processing branch, the depth image processing branch, and the feature fusion and angle estimation module.

The surface EMG signal processing branch consists of two main operations: data preprocessing and feature extraction. The preprocessing stage employs standard data filtering techniques to mitigate distributional changes caused by the non-stationarity of sEMG signals. A time-triggered windowing method synchronizes sEMG signals with the lower-rate depth image data and provides multi-scale raw data channels to generate more reliable feature vectors for the training process. An encoder–decoder framework with shared weights provides a higher level of feature compression vectors for angle estimation.

Considering the lower data acquisition rate of the RGB-D camera compared to the sEMG equipment, a synchronization trigger signal is created for each frame. Using 3D information provided by the RGB-D camera, a CNN-based feature extraction block simultaneously captures spatial characteristics of 3D human joint positions.

To adapt to different dimensions and scales of the extracted features, the proposed framework employs a feature fusion block through a series of feature concatenations. To expedite and enhance the effectiveness of information acquisition, multiple down-sampling and pooling layers are introduced. The final joint angle regression is performed using an LSTM network.

### 2.2. sEMG Signal Processing

#### 2.2.1. Preprocessing

The noise in sEMG signals can significantly affect the process of data acquisition and feature extraction. Considering the baseline energy density spectrum of EMG signals, band-pass filters are applied to eliminate noise and artifacts. Due to the potential loss of information when standardizing the data sequence, no additional preprocessing is applied to the raw data.

Typically, sEMG signals are segmented using a sliding window to obtain multiple samples over different time intervals, but in our case, the sliding window may increase the complexity of data asynchrony. Triggered by the acquisition of a depth image, a timestamp is generated to create a series of fixed windows for sEMG signals, each containing a different length of sEMG signal. The varying window lengths provide different timescales of digitized sEMG data, enhancing the diversity and redundancy of the sEMG signal information.

**Figure 2:**
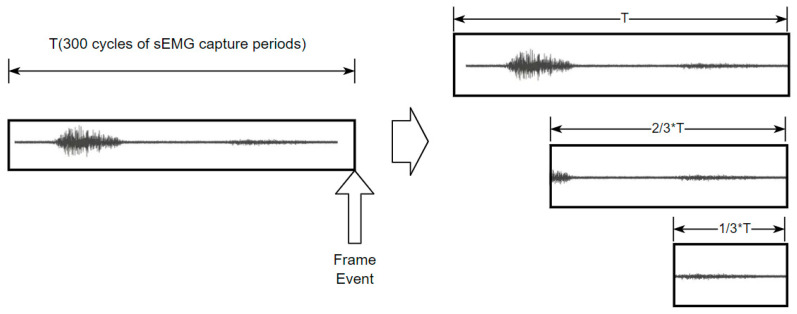
Windowing different scales.

As shown in Figure 2, each frame captured by the RGB-D camera generates a time-triggered event, and this timestamp automatically forms a base window for each sEMG channel. The interval of the base window is fixed to 300 cycles of sEMG capturing periods. To achieve different scales of sEMG signals, processing windows are refactorized to 3 scales, and each signal scale ends precisely when the depth image is captured with a duration of T, $\frac{2}{3}T$, and $\frac{1}{3}T$.

#### 2.2.2. Multi-Scale Feature Extraction Block

The convolutional neural network (CNN) in feature extraction of sEMG signals has been verified as an efficient and robust method. Based on the CNN structure and the capabilities of the autoencoder network, this paper proposes a novel feature extraction block to handle multiple scales of digitized sEMG data, yielding more compressed and reconstructive information. The reconstruction of the sEMG image can better adapt to varying data distributions in raw sEMG data by utilizing reconstruction error. The encoder–decoder network has proven effective in extracting higher-dimensional features. We redesigned the network to be more sensitive to differently scaled windows of sEMG data by using shared weight vectors. By updating shared weights, the extraction block maintains both training efficiency and information sensitivity.

**Figure 3:**
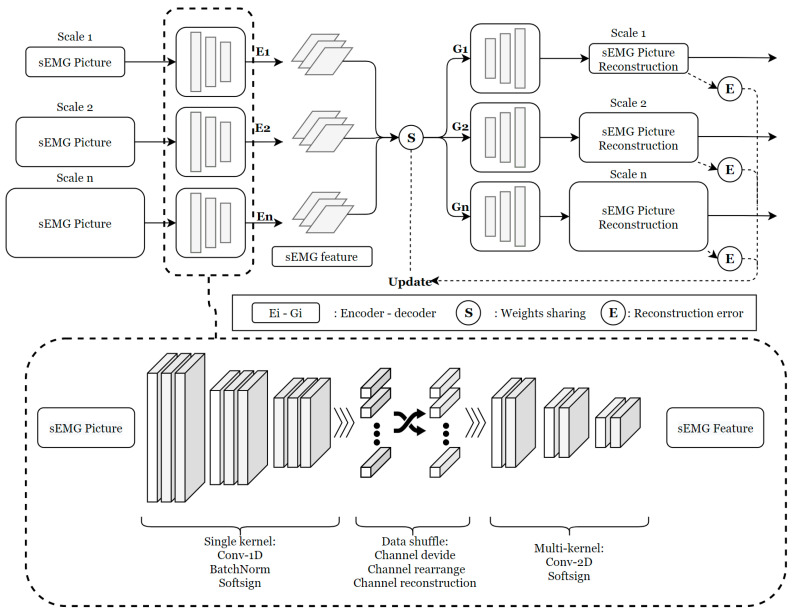
Multi-scale processing and updating.

As shown in Figure 3, separate encoders and decoders are applied to each sEMG data channel. The encoder and decoder have symmetric architectures with feature outputs, although their corresponding parameters differ. The input of the encoder network is a serial combination of different channels of sEMG signals, each representing a different source of signal. Depending on the varying timescales of the sEMG data sequences, different network parameters are applied. Using the horizontal and vertical indices of the sEMG data, the discrete sEMG data are combined in the form of an image. The convolutional layers of the encoder consist of single-kernel and multi-kernel components. The single-kernel component compresses only the temporal information, while the multi-kernel component applies 2D convolution to a mixture of temporal and spatial information. A shuffle technique is applied between the single-kernel and multi-kernel components to capture the interrelationships and interactional impacts between related muscle groups.

### 2.3. Vision-Based Feature Extraction

To estimate human body joint angles, the processing system should construct a skeleton model that represents human body posture using the appearance in the image. In this paper, we use a human pose estimation method based on a convolutional neural network (CNN) using depth images acquired from an RGB-D camera. Although depth images exhibit less detailed information than optical images, the feature extraction block faces a trade-off between spatial reliability and locational precision of image features. By eliminating background noise and lighting effects through 3D imaging techniques, a more accurate silhouette of the human body can be extracted from the depth image. The idea is to employ a series of small, lightweight networks to perform feature extraction for depth images. The initial convolutional block uses small kernel sizes, with batch normalization and ReLU applied after each convolutional layer. Several convolutional layers are combined to form a residual block. We also implemented average pooling layers at the beginning, middle, and end of the feature extraction block.

The aim of the depth image feature extraction block is to form a high-precision heatmap of joint 3D locations. The feature vectors are then delivered to the data fusion block along with multi-scale sEMG features. 

### 2.4. Data Fusion and Angle Estimation

**Figure 4:**
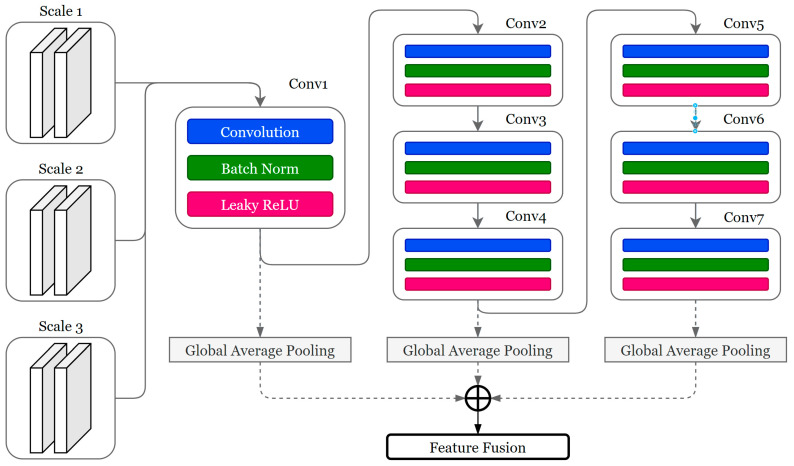
Feature fusion network.

To obtain a reliable and stable outcome from the estimation network, attention must be paid to both global and local information. Motivated by the architecture of the feature pyramid, we designed a fusion network as shown in Figure 4. The input of the data fusion block is a concatenation of sEMG features and depth image features. Extracted image features are copied to each scale of the sEMG feature vector. The combined data are subjected to a series of down-sampling operations. After four iterations of the convolutional layer with a stride of 2, the number of channels doubles each time. The convolutional layer consists of three parts: a convolutional layer with a 3 × 3 kernel and padding of 1, batch normalization, and Leaky ReLU. To account for the loss of superficial semantic information in the first four layers, three global average pooling layers are applied to the outputs of the 1st, 4th, and 7th convolutional layers to generate the final fused feature map. As multi-layered semantic information is formed by three feature vectors, concatenation is applied to obtain the fused feature map of sEMG signals and depth images.

To leverage the strong temporal correlation among joint angles, sEMG signals, and visual signals, we fed the fused features into an LSTM network for joint angle prediction. LSTM is well suited to capturing the dynamic characteristics of time-series data and can more effectively handle multimodal temporal features. This approach improves the model’s generalization ability and leads to enhanced performance.

### 2.5. Experimental Environments

Since no current public dataset contains simultaneously captured data of sEMG signals and depth images of body movements, we created a custom dataset using sEMG signal capture equipment and an RGB-D camera, with motion capture system markings. To evaluate the performance of the proposed framework, we performed a horizontal analysis between our method and state-of-the-art methods on our custom dataset to conduct the following experiments and achieve corresponding results.

1. We compared single-modal sensor capture and processing of sEMG signals and RGB-D camera data. By highlighting the disadvantages of a single-sensor system, we demonstrated the improved accuracy of joint angle estimation using the combination of sEMG signals and RGB-D camera data.
2. Considering subject diversity, we performed cross-comparisons between different subjects and analyzed the errors by accounting for scenario effects and human behavior effects.
3. Noting that ground obstacles may influence visual data capture, we introduced a series of occlusions in our test of knee angle prediction.
4. We compared a series of state-of-the-art methods with ours using our dataset and analyzed the performance from different perspectives.

Under laboratory experimental conditions, the hardware equipment used in this study includes the following:

- Computer: Windows 10 64-bit (DELL, Beijing, China)and Ubuntu 18.04.6 LTS 64-bit double operating system, carrying a CPU of Intel^®^ Core™ i7-8700K with 16 GB RAM and an Nvidia GEFORCE GTX 1660 Ti graphics processing card.
- RGB-D camera: Intel RealSense Depth Camera D435i with specifications of 1920 × 1080 colored resolution and 1280 × 720 depth resolution @30 fps, 69° horizontal and 42° vertical field-of-view wide-angle lens.
- sEMG signal capture equipment: Delsys wearable sensors with analog output in range of 11 mV and sampling rate of 1111 Hz. The average noise of overall channel is inferior to 3 uV RMS @ 10–850 Hz.
- Motion capture equipment: VICON motion capture system with 1.3 MP resolution @ 250 Hz. The capture system has 98.1° horizontal and 50.1° vertical field of view.

#### 2.5.1. Participants

Motivated by the hand gesture dataset created by putEMG [38], we created a dataset containing a total of 12 participants aged 24 to 32 years. The average height of our participants is 173.8 ± 6.2 cm, and the average weight is 72.5 ± 8.9 kg. None of the participants had motor dysfunction. Participants were instructed to walk on a treadmill at a constant speed of 4.5 km/h with a zero-degree incline. Each participant completed the procedure eight times, ensuring that the movements of the hip, knee, and ankle joints were consistently and accurately captured by the camera. 

This study was conducted in accordance with the Declaration of Helsinki. Written informed consent was obtained from all participants.

#### 2.5.2. Data Acquisition

**Figure 5:**
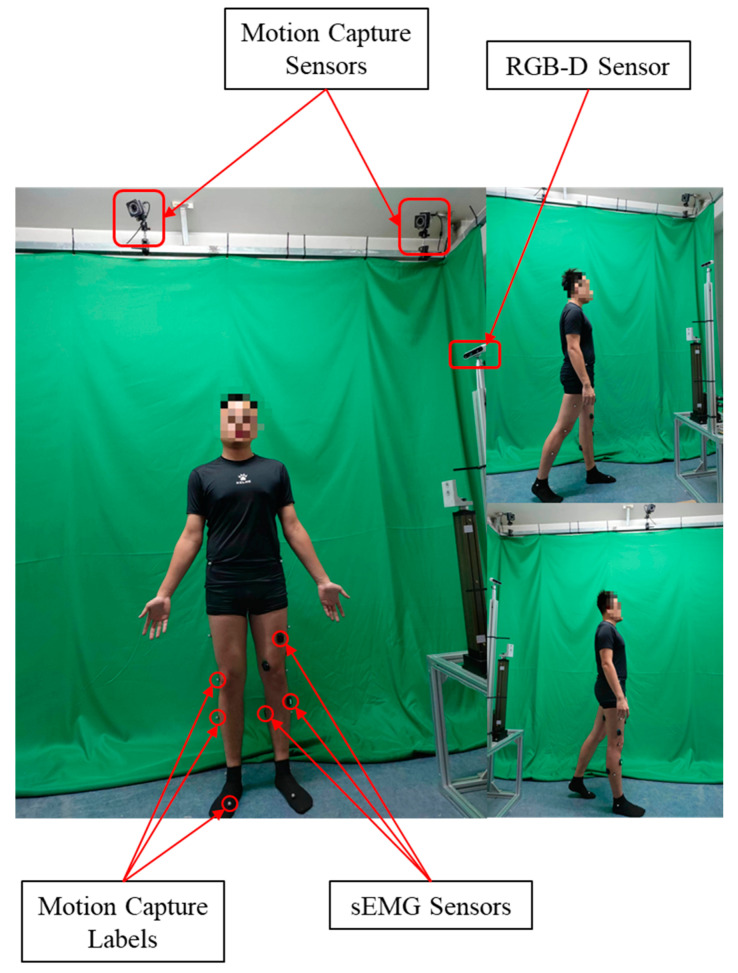
Dataset capture environment.

The main goal of establishing the dataset is to obtain synchronized multi-channel sEMG signals and RGB-D camera images, the dataset capture environment is illustrated in Figure 5. Due to the low latency and high accuracy of the motion capture system, we utilized the VICON motion capture system, as mentioned above, to establish ground truth for human joint angles. Under laboratory conditions, we established the hardware setup as follows. In this study, a total of nine sensors were used to monitor the lower limb muscle groups. These muscles include the rectus femoris (RF), vastus medialis (VM), vastus lateralis (VL), tibialis anterior (TA), soleus (SOL), semitendinosus (SEM), biceps femoris (BF), medial gastrocnemius (MG), and lateral gastrocnemius (LG). The placement of the sensors was approximate and adjusted through palpation and EMG recordings for different subjects. The skin of each subject was cleaned with rubbing alcohol prior to electrode placement to ensure optimal conditions.

A crucial aspect of data acquisition is the synchronization of the depth video stream and the sEMG signal stream. The capture moment of each depth image can be used as a time stamp to generate a synchronization signal. To achieve real-time performance in the data acquisition system, threading techniques were implemented by creating parallel tasks consisting of a system-level hard real-time timer, an image capture task, and a multi-channel sEMG capture task. Maximizing the utilization of system RAM improves performance by reducing latency between sEMG signals and image capture events through pipelined procedures for storing data in RAM and writing data to the hard disk.

## 3. Results

**Figure 6:**
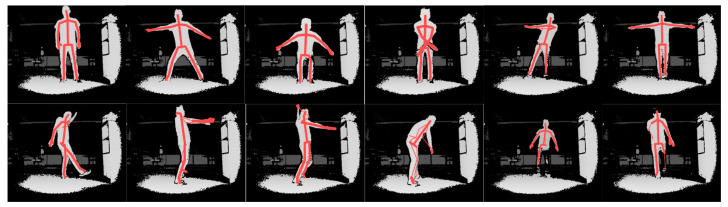
Depth camera-processed data: the upper row shows the correct recognition scenarios in short (1.5 m) range and long range (3 m); the lower row shows the misrecognition scenarios.

Through a single depth image containing the 3D locational information of each pixel, we can relatively easily reconstruct the human body with 3D joint locations; however, the consistency and stability of the regression output cannot be guaranteed. Vision data processing allowed us to extract 19 human body joint 3D locations. The data were recorded indoors with a single person at two different ranges. Considering the application scenario, participants were recorded from different angles. As shown in Figure 6, the joints could be accurately extracted when facing the depth camera and in the absence of occlusion. Despite the accuracy of joint locations, different angles significantly affect the feature extraction stage, leading to misrecognitions and altering the relationships between neighboring joints. To estimate the prediction error with occlusions, we conducted predictions using blocked inputs in our experimental series.

The joint angles were calculated from 3D locations. We used a convolutional neural network on depth images [39] to calculate the knee angle during walking. Conversely, we used a BP neural network [40] on sEMG signals to obtain knee angles. Using 10 sets of unrelated data, we obtained three averaged curves of knee angle variation over time.

**Figure 7:**
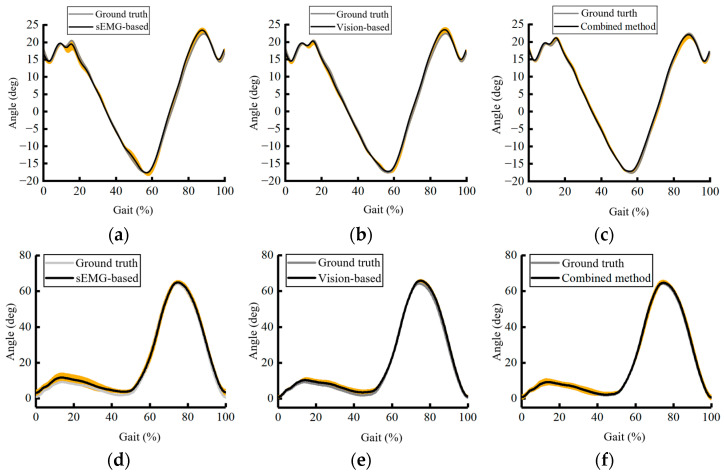

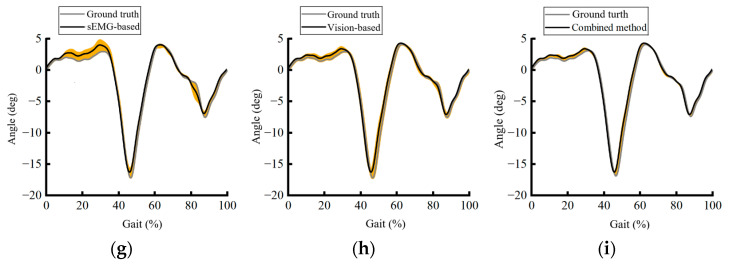
Knee angle, hip angle, and ankle angle prediction while walking on the treadmill based on different methods, the yellow shaded area represents the variance of the estimation results: (**a**) sEMG-based hip angle prediction; (**b**) vision-based hip angle prediction; (**c**) combined hip angle prediction; (**d**) sEMG-based knee angle prediction; (**e**) vision-based knee angle prediction; (**f**) combined knee angle prediction; (**g**) sEMG-based ankle angle prediction; (**h**) vision-based ankle angle prediction; (**i**) combined ankle angle prediction.

Figure 7 presents the predictions corresponding to different algorithms, with the gray curve representing the standard knee joint angle results computed by VICON. The average maximum prediction error for the sEMG-based methods is 2.72 ± 1.32°, while for the visual methods, it is 2.67 ± 0.69°. The fusion of visual and EMG-based methods results in a lower prediction error of 1.04 ± 0.52°.

**Figure 8:**
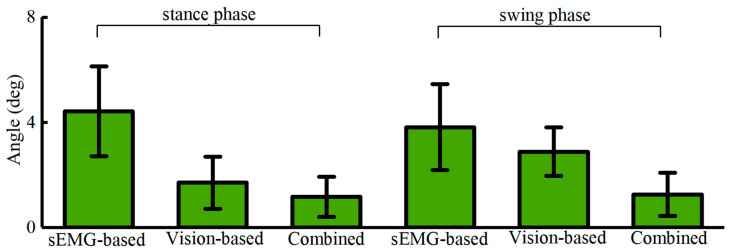
Knee angle predictions via different participants.

Individual variations may cause significant differences in predictions; therefore, we applied different datasets with varying distributions. During the stance phase, the sEMG-based method achieved a prediction error of 4.42 ± 1.71°, the vision-based method achieved a prediction error of 1.71 ± 0.99°, and the combined method achieved a prediction error of 1.17 ± 0.76°. During the swing phase, the sEMG-based method achieved a prediction error of 3.82 ± 1.63°, the vision-based method achieved a prediction error of 2.89 ± 0.93°, and the combined method achieved a prediction error of 1.26 ± 0.82°. To assess the model’s adaptability, we also validated the hip and ankle joints, and the results are presented in Figure 8 and Table 1 below.

By combining sEMG signals and vision signals, we achieved promising results with partial blocks. To simulate obstacles on the ground that may cause loss of visual capture of the ankles, we implemented blocks of four different heights.

**Figure 9:**
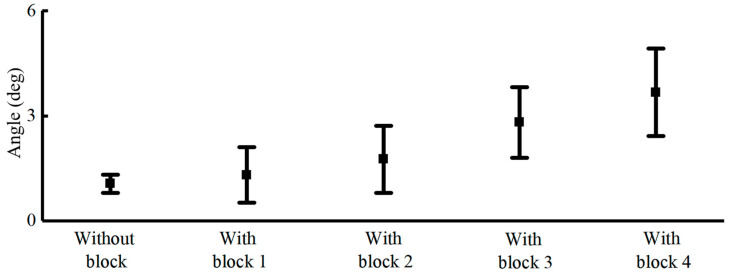
Knee angle prediction with partial blocks.

Figure 9 presents knee angle predictions with four different blocks implemented at the ankle. Without blocks, we achieved a prediction error of 1.04 ± 0.26°, while with increasing obstacle height, the mean errors rose from 1.31 degrees to 3.67 degrees, with variances ranging from 0.79 degrees to 1.25 degrees.

Using deep belief networks [41] and long short-term memory [42,43], we calculated the root mean square errors for each method. As shown in Table 2, our method achieves significant improvements compared to baseline methods.

## 4. Discussion

In knee angle prediction, both surface electromyographic (sEMG) and visual methods can achieve a certain level of accuracy. These results indicate an inherent correlation between EMG, visual information, and joint angles. This implicit mapping relationship can be constructed using an end-to-end approach.

Compared to visual methods, EMG-based methods show inferior performance in both mean and variance. The average maximum prediction error for EMG-based methods is 2.72 ± 1.32°, while for visual methods, it is 2.67 ± 0.69°. On the one hand, this is because EMG signals are inherently related to motion, making their relationship less explicit compared to direct visual observation. On the other hand, EMG signals are characterized by high-amplitude noise, which can decrease algorithm performance due to interference. The maximum error for EMG-based methods occurs during the stance phase of the lower limb, where significant muscle vibrations increase EMG signal noise, leading to higher prediction errors. In contrast, during the relatively stable swing phase, EMG-based methods yield better results. For visual-based predictions, the maximum error occurs during the swing phase due to continuous changes in multiple limbs and background variations, impacting predictions and decreasing performance. Pure visual methods exhibit good stability and strong result repeatability. The fusion of visual and EMG-based methods effectively compensates for the shortcomings of both, leading to improved accuracy with a maximum angle error of 1.04 ± 0.52°.

In practical applications, data often have distributions different from our training set. When the distributions of training and test data differ, prediction errors increase. Particularly in EMG-based methods, individual differences significantly impact predictions, as variations in EMG distribution due to individual differences lead to a rapid increase in errors. Conversely, methods based on visual information are relatively less affected by individual differences. Furthermore, relationships between sparse sEMG signals of cooperative muscles can be extracted using multi-scale feature maps.

Similar phenomena were observed in further experiments on hip and ankle angle predictions. However, we observed a slight increase in variation in ankle angle predictions, which may be due to the flexibility of movement patterns. Our model is designed to combine sEMG signal feature maps and vision features to address this issue, but the insufficient resolution of the RGB-D camera introduces drifting of foot posture. The results from ablation experiments show significant improvements using the combined method.

Ground obstacles can cause feature loss in visual capture, leading to prediction errors. To test the robustness of our prediction model, we introduced occlusions in the knee angle prediction scenario. Four categories of occlusions were introduced in this research, and as the level of occlusion increased, the prediction error for EMG-based methods also increased gradually. Notably, in occlusion category 1, which involves relatively low occlusion height, the visual impact was minimal. Overall, prediction error with the highest level of occlusion still shows better performance compared to the EMG-based method.

Compared to joint angle prediction methods based on sEMG signals using DBN and LSTM, our method demonstrated more competitive results in our test cases as shown in Table 2. By incorporating visual depth data, the spatial positioning of joints becomes more precise. Higher-dimensional data sequences, such as depth image data, offer a more robust representation of human skeletal postures. However, 3D imaging techniques typically require more time for sensing and preprocessing, which leads to temporal sparsity in the data sequence. In critical real-time HMI scenarios, the reaction and adjustment phases of the decision–reaction cycle are not as competitive due to the slower capture cadence of 3D imaging data. For this reason, human exoskeleton control benefits from the use of high-frequency sensors like sEMG and IMU, which provide faster and more frequent data. By combining sEMG signals and depth image data to extract distinct physical features, we developed a muscle–skeleton model that establishes a temporal–spatial link, enabling a feature fusion pattern for more accurate movement prediction. By incorporating 3D visual data as spatial markers and calibration references within the proposed framework, the robustness and accuracy of lower limb joint angle predictions are significantly improved. 

In future work, we plan to focus on the long-term stability of our proposed human joint angle prediction method, ensuring that it remains robust and reliable over extended periods and across various conditions. This will involve continuous refinement of the algorithm to adapt to real-world challenges, such as signal drift and variations in sensor data. Additionally, we aim to implement our method in exoskeleton systems, where accurate joint angle estimation is critical for providing responsive and precise assistance to users. By integrating our approach into exoskeletons, we hope to enhance their effectiveness in applications such as rehabilitation and mobility support, ultimately contributing to improved user outcomes.

## 5. Conclusions

In this paper, we propose a novel dual-branch network framework for estimating human joint angles, leveraging the strengths of both sEMG signals and RGB-D camera data. By integrating a convolutional autoencoder for high-level sEMG feature extraction with a vision-based joint regression network, the proposed method addresses the challenges associated with non-stationary sEMG signals and the limitations of vision-based approaches, such as latency and shading. The enhanced feature extraction and fusion pipeline effectively combines multi-scale sEMG and RGB-D data, resulting in a reliable and accurate estimation of human joint angles. The proposed framework not only advances the field of motion analysis and gesture recognition but also demonstrates the potential for more precise motion intention prediction by overcoming the inherent challenges of sEMG signal variability and vision data acquisition.

## Figures and Tables

**Table 1:** Estimation errors in degrees for hip angle, knee angle, and ankle angle via different participants.

		sEMG-Based	Vision-Based	Combined
Hip	Stance phase	3.97 ± 1.35	2.15 ± 0.87	1.47 ± 0.63
	Swing phase	3.64 ± 1.11	2.57 ± 0.86	1.89 ± 0.67
Knee	Stance phase	4.42 ± 1.71	1.71 ± 0.99	1.17 ± 0.76
	Swing phase	3.82 ± 1.63	2.89 ± 0.93	1.26 ± 0.82
Ankle	Stance phase	4.21 ± 1.45	2.23 ± 0.89	1.52 ± 0.65
	Swing phase	3.97 ± 1.18	2.98 ± 0.89	2.12 ± 0.55

**Table 2:** Comparisons of RMSE between different methods.

	DBN [41]	LSTM [42]	LSTM [43]	Ours
Hip	3.58	1.31–2.48 *	1.33	0.68
Knee	3.96	1.37–1.74 *	2.16	1.68
Ankle	2.45	1.31–1.56 *	1.73	0.73

* The results are calculated in three phases; we note the RMSE range in the table.

## Data Availability

The data are available from the corresponding author on reasonable request.

## Abbreviations

The following abbreviations are used in this manuscript:

HMI	Human–machine interaction
sEMG	Surface electromyography signal
FBG	Fiber Bragg grating
IMU	Inertial measurement unit
CNN	Convolutional neural network
RGB-D	Red green blue and depth
RAM	Random-access memory
